# Retraction: COP9 signalosome CSN4 and CSN5 subunits are involved in jasmonate-dependent defense against root-knot nematode in tomato

**DOI:** 10.3389/fpls.2025.1666310

**Published:** 2025-07-30

**Authors:** Frontiers Editorial Office

The journal retracts the article cited above.

Following publication, the authors contacted the Editorial Office to request a correction to the cited article. This raised concerns regarding the validity of the data in the article. The authors failed to provide all the raw data or a satisfactory explanation during the investigation, which was conducted in accordance with Frontiers’ policies. Given the concerns, and the lack of raw data, the editors no longer have confidence in the findings presented in the article.

This retraction was approved by the Chief Executive Editor of Frontiers. The authors received a communication regarding the retraction and had a chance to respond. This communication has been recorded by the publisher.

